# The Mechanical Effect of MnO_2_ Layers on Electrochemical Actuation Performance of Nanoporous Gold

**DOI:** 10.3390/nano10102056

**Published:** 2020-10-18

**Authors:** Zhifei Han, Zhengpan Qi, Qiang Wei, Qibo Deng, Ke Wang

**Affiliations:** 1 Tianjin Key Laboratory of Advanced Functional Porous Materials, Institute for New Energy Materials & Low-Carbon Technologies, School of Materials Science and Engineering, Tianjin University of Technology, Tianjin 300384, China; 183124331@stud.tjut.edu.cn; 2 Research Institute for Structure Technology of Advanced Equipment, School of Mechanical Engineering, Hebei University of Technology, Tianjin 300401, China; zhengpan_qi@hebut.edu.cn (Z.Q.); weiqiang.tju@163.com (Q.W.); 3 Key Laboratory of Advanced Energy Materials Chemistry (Ministry of Education), College of Chemistry, Nankai University, Tianjin 300071, China; 4 School of Materials Science and Engineering, University of Shanghai for Science and Technology, Shanghai 200093, China

**Keywords:** electrochemical actuation, MnO_2_ covering, nanoporous gold, actuation-potential response, mechanical effect

## Abstract

This study investigated the electrochemical actuation behavior of nanoporous material during the capacitive process. The length change of nanoporous gold (npg) was in situ investigated in a liquid environment using the dilatometry technique. The mechanical effect of MnO_2_ layers was introduced in this work to improve the actuation characteristics of the npg samples. Our work found that the actuation behavior of npg sample could be significantly modulated with a covering of MnO_2_ layers. The electrochemical actuation amplitude was efficiently improved and strongly dependent on the thickness of MnO_2_ layers covered. Aside from the amplitude, the phase relation between the length change and the electrode potential was inverted when covering the MnO_2_ layer on the npg samples. This means the expansion of the npg samples and the contraction of samples covered with the MnO_2_ layer when electrochemical potential sweeps positively. A simple finite element model was built up to understand the effect of the MnO_2_ layer. The agreement between the simulation result and the experimental data indicates that the sign-inverted actuation-potential response of nanoporous gold contributes to the mechanical effect of MnO_2_. It is believed that our work could offer a deep understanding on the effect of the MnO_2_ layer on the electrochemical actuation and then provide a useful strategy to modulate the actuation performance of nanoporous metal materials.

## 1. Introduction

An electrochemical actuator can directly convert the electrical energy to mechanical deformation during electrochemical processes, which has gained increasing interest in the fields of nanomaterials. The electrode materials of electrochemical actuators could be carbon nanotubes [1,2,3], conducting polymers [4,5], transition metal dichalcogenides [6,7], metals [8,9], and ionic polymer-metal composites [10,11]. In contrast to conventional actuator materials, metallic muscles have much better conductivity and mechanical properties (e.g., stiffness, and strength). In electrochemical actuator materials, the bulk volume not only plays a critical role for actuating, but also the interface surface area [12,13,14,15,16,17,18]. By employing dealloyed nanoporous metals with high surface-area-to-volume ratios, the new actuators represent a novel class of smart materials undergoing reversible dimensional changes upon the injection of an electronic charge in the space-charge region at the nanoporous metal–electrolyte interface [8,19]. When using nanoporous metals as actuator electrodes, the reversible actuation can be induced by a low applied voltage, normally on the order of ~1 V, and the induced amplitude is comparable to those of commercial piezo-ceramics (~0.2%). Moreover, nanoporous metals in bulk form can also undertake considerable reversible compressive loading, which is considered as a pre-requisite for actuator applications. A number of new actuators based on nanoporous metals have been recently prepared and reported as desirable artificial metallic materials for the fast response and high performance of electrochemical actuation in the past decade [19,20,21,22,23,24,25,26,27,28,29,30,31,32,33]. One of most efficient strategies to improve the actuation behavior is to prepare a nanoporous structure with different metals (e.g., Au, Pd, AuPt, Ag, Al, Ni) [19,20,21,22,23,24,25,26,27,28,29,30]. The electrochemical actuation of nanoporous metal materials is also strongly dependent on the electrochemical process (e.g., surface oxide) [31], which can be used as a tool to tune the actuation behavior. Shi et al. introduced the hydrogen electrosorption to modulate the actuation of nanoporous palladium [32]. Zhang and Jin reported an interesting strategy from the viewpoint of the structural composite to improve the linear actuation of nanoporous gold (npg) by forming Au/npg multilayer composites [33]. More and more new-style efficient strategies for the improvement in the electrochemical actuation of nanoporous metals are still under exploration and investigation [34,35,36].

In this work, we introduce a common supercapacitive material, MnO_2_, using the electrochemical deposition technique to improve the actuation behavior of nanoporous metal materials. Since the electrochemical actuation performance of nanoporous gold has been well-investigated in the past decade and the sample preparation process is standard [19,20,21,31,33,34,35], this work chose nanoporous gold as a typical case to study the strategy. Our work found that the deposited MnO_2_ thin layer could change the electrochemical actuation phase of npg (i.e., from expansion to contraction) as well as the actuation magnitude. A simple finite element model was also built in our study to understand the effect of the MnO_2_ layer on the actuation behavior of npg. This work offers a simple method to modulate the actuation behavior of nanoporous metal materials in response to electrochemical potentials.

## 2. Materials and Methods 

Parent alloys of Au_25_Ag_75_ were prepared by repetitive arc melting of Au and Ag wires (purity 99.99%) under an argon atmosphere. The nanoporous gold (npg) samples in use were prepared by electrochemical dealloying under potentiostatic control (AUTOLAB PGSTAT302N) in 1 M HClO_4_ in a three-electrode electrochemical cell. The detail about the preparation of npg can be found in previous reports [10,31,33,34,35]. The reference electrode used in this study was a pseudo Ag/AgCl electrode, whose potential with respect to the standard hydrogen electrode (SHE) was 0.53 V [35]. 

**Figure 1:**
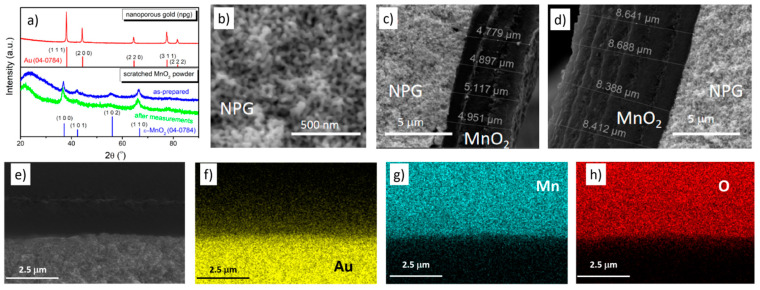
(**a**) The XRD pattern of npg and scratched MnO_2_ powder. The SEM images of different samples: (**b**) nanoporous gold (npg); (**c**) npg covered with MnO_2_ layer after three electrochemical deposition cycles (label: npg+3MnO_2_); (**d**) npg covered with MnO_2_ layer after six electrochemical deposition cycles (label: npg+6MnO_2_); (**e**) the interface of npg sample and MnO_2_ layer; (**f**–**h**) elements mapping images.

The MnO_2_ layers were electrochemically deposited onto the outer surface of npg in a 0.1 M Na_2_SO_4_ + 5 mM MnSO_4_ from 0 to 1 V (vs. pseudo Ag/AgCl electrode) at a potential scan rate of 0.5 mV s^−1^. The corresponding samples after electrochemical deposition was named as npg + *x* MnO_2_ (*x* is the cycles of MnO_2_ deposition). The X-ray diffraction (XRD) pattern revealed that the obtained MnO_2_ material in an electrochemical deposition manner was ε-MnO_2_ (see Figure 1a), consistent with the previous reports [37]. The crystal structure did not change after applying the electrochemical potentials.

All samples in this study were investigated under the same measurement conditions for the characterization of electrochemical actuation behavior. The actuation performance of samples was investigated by means of in situ dilatometry with a focus on the length change in response to the potential variations during cyclic voltammogram in a 0.1 M Na_2_SO_4_ aqueous solution. The dilatometer (LINSEIS L75, Selb, Germany) was equipped with an electrochemical cell and the constant pressure of 100 mN was imposed on the sample. The sample size for in situ dilatometry experiment was 1 × 1 × 2 mm^3^. 

The morphology of samples was characterized by a scanning electron microscope (SEM, Quanta FEG 250, Hillsboro, OR, USA). The crystal structures were characterized by an X-ray diffractometer (XRD, Rigaku D/max-2500, Tokyo, Japan) with Cu Kα radiation.

## 3. Results and Discussion

The SEM image in Figure 1b exhibited the typical morphology of nanoporous gold (npg) with a ligament size of ~25 nm. The SEM images in Figure 1c,d show that the MnO_2_ layer was about 5 μm thick after three cycles of electrochemical deposition and the thickness was about 8.5 μm after six deposition cycles. The element mapping images in Figure 1e–h indicate that the deposited MnO_2_ layer was covered round the circumference of the npg bulk material.

**Figure 2:**
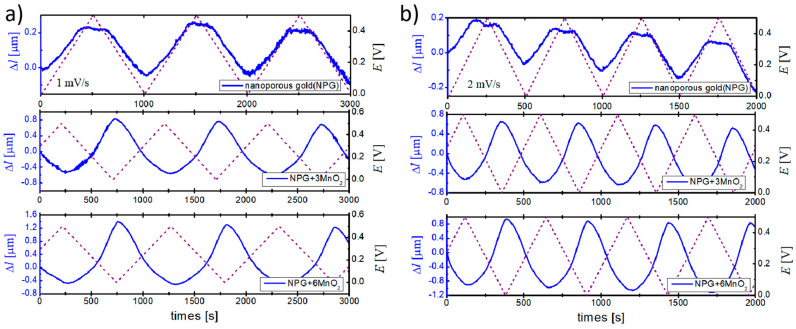
The length change (blue solid line) of three different samples (npg, npg+3MnO_2_, and npg+6MnO_2_) in response to the change in electrochemical potential (dotted line) in the time domain at a potential scan rate of 1 mV s^−1^ (**a**) and 2 mV s^−1^ (**b**).

The actuation behavior of different samples (npg, npg+3MnO_2_, npg+6MnO_2_) was investigated in a time domain using an in situ dilatometry technique when the potential was swept in the double layer region between 0 and 0.5 V (versus the home-made Ag/AgCl reference electrode). The results recorded at potential scan rates of 1 mV s^−1^ and 2 mV s^−1^ are shown in Figure 2. The typical actuation behaviors of the free-standing monolithic npg (in Figure 2) showed that the npg sample expanded during anodic potential sweeping and contracted when the sweeping direction was reversed. The phase between the applied electrode potential and corresponding length change was close to 0 degrees. The result is in agreement with the literature [33]. After deposition of the MnO_2_ layer on the macroscopical surface, both npg+3MnO_2_ and npg+6MnO_2_ exhibited the opposite variation trend with potential change in comparison with the pure npg sample; namely, the length change of these two samples were contracted during anodic potential sweeping. The phase between the applied electrode potential and corresponding length change was inverted to 180 degrees. Aside from the phase change, it can be seen that there was a larger amplitude of potential-induced actuation with the thicker MnO_2_ layer. Figure 2b shows that the actuation behaviors of the npg and npg+MnO_2_ composite at 2 mV s^−1^ was similar to that measured at 1 mV s^−1^.

**Figure 3:**
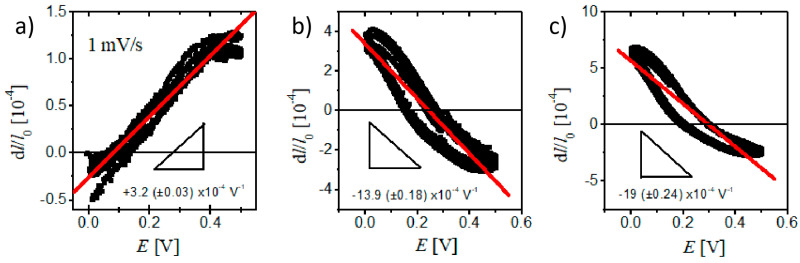
The actuation response as the function of electrode potential at a scan rate of 1 mV/s for npg (**a**), npg+3MnO_2_ (**b**), and npg+6MnO_2_ (**c**). The best linear fit is shown as red lines in the figures.

Considering the original length of the npg sample (2 mm) in this work, the relation between the length strain and electrode potential was plotted in Figure 3. The best linear fitting gave the slope value of +3.2 (± 0.03) × 10^−4^ V^−1^ for the npg sample whereas the slope values were −13.9 (± 0.18) × 10^−4^ V^−1^ and −19 (± 0.24) × 10^−4^ V^−1^ for npg+3MnO_2_ and npg+6MnO_2_, respectively. The covered MnO_2_ thin layer could significantly change the electrochemical actuation direction as well as the magnitude of the npg in response to the potential variation.

**Figure 4:**
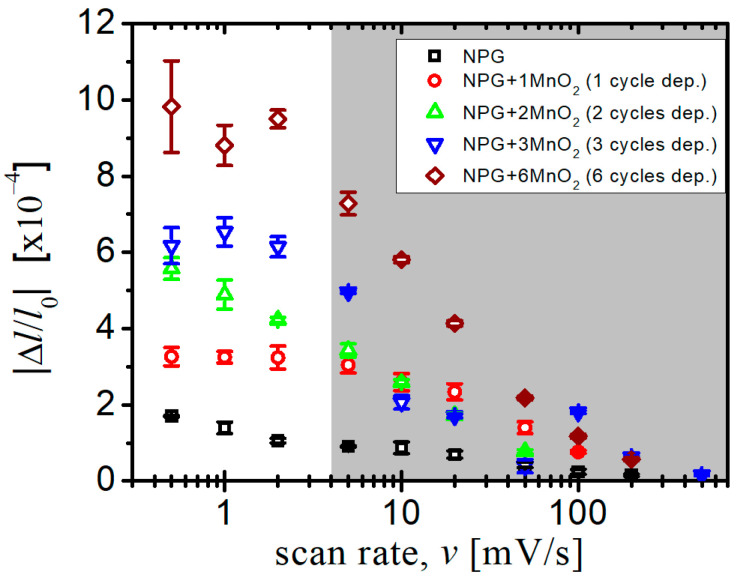
The actuation amplitude of different samples as the function of potential scan rate.

A study of the intermediate thickness of the deposited MnO_2_ layers (Figure 4) was conducted at different potential scan rates. The data revealed the trend of actuation amplitude in Figure 2 and Figure 3 to be systematic. For all samples, the actuation amplitude increased with the decrease in the potential scan rate. This can be attributed to the slow ion transportation from the electrolyte to ligament surface at a fast scan rate.

**Figure 5:**
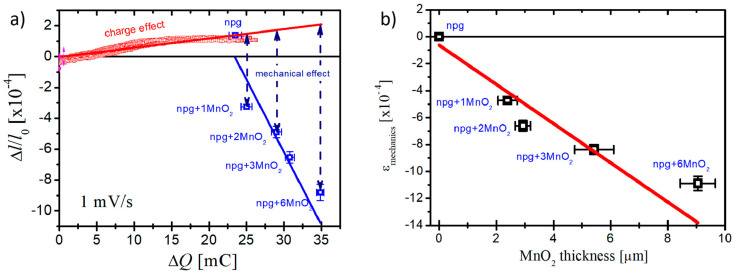
The actuation response of different samples as the function of the charge change (**a**) and the mechanical effect of MnO_2_ on the actuation behavior of nanoporous gold (**b**).

In recent years, several nonlocal continuous models have been developed for the evaluation of strains when considering the nanostructure [38,39,40], which then provide a mathematically well-posed and technically reliable methodology to assess the scale effects in the nano-structures. Barretta et al. reported the modified nonlocal strain gradient elasticity for nano-rods and its application to carbon nanotubes considering the size effect [40]. We analyzed the effect of the covered MnO_2_ on electrochemical actuation performance of nanoporous gold. Based on the literature about the electrochemical actuation of nanoporous metals, the direct link between macroscopical strain and charge induced by electrochemical potential can be shown in a fundamental expression as [17,22,41]:

(1) $$\frac{\Delta l}{l_{0}}=-\frac{2\rho }{9Km}\Delta f=-\frac{2\rho \Delta Q}{9Km}\varsigma =-const.\left(\Delta Q\varsigma_{\mathrm{Au}}+f_{{\mathrm{MnO}}_{2}}\right)$$

where *K* denotes the bulk modulus of the metal; *ρ* is the density of the solid phase; and *m* is the sample mass. The parameter ς describes the coupling strength between the surface mechanics and the electrode process and can be quantified by two independent measurements: (i) from the variation of electrode potential (*E*) with surface strain (*ε*) [12,13,14,42,43], and (ii) from the variation of surface stress (*f*) with charge density (*q*) [15,16,17]. The negative-valued ς reported in the electrochemical capacitive process revealed that positive charge induced the expansion of nanoporous gold [31,33]. Since the thickness of MnO_2_ (~9 μm) was much less than that of the npg bulk sample (1 mm), the change in the material properties (*ρ*, *K*, *m*) was neglected. Thus, the effect of MnO_2_ on the actuation of npg can be considered as the charge state (Δ*Q*) and mechanical coupling factor (*f*). Figure 5a plots the actuation strain as the function of charge change for different samples. In the case of the npg sample, there was a positive linear relation between the actuation strain and charge state, which means more positive charge and more expansion. After deposition of the MnO_2_ layer, the samples contracted with positive charge. This indicates that the change in the actuation behavior of npg is not due to the charge change when covering the MnO_2_ layer. The contraction amplitude seems linear with the cycles of MnO_2_ deposition. The mechanical effect of the deposited MnO_2_ on the actuation of npg can be obtained by excluding the effect of charge from the actuation amplitude and the data are shown in Figure 5b. The contraction amplitude becomes larger with the thicker MnO_2_ layer and the best linear fit gave the slope as −1.45 (± 0.25) × 10^−4^ μm^−1^. 

The charge can be stored in the MnO_2_ layer by the reversible redox reaction in a neutral electrolyte (Na_2_SO_4_) during the electrochemical charge and discharge process according to Equation (2) [37]:

(2) $${\mathrm{MnO}}_{2}+{\mathrm{Na}}^{+}+e^{-}\overset{\mathrm{chaerge}/\mathrm{discharge}}{↔}\mathrm{MnOONa}$$

**Figure 6:**
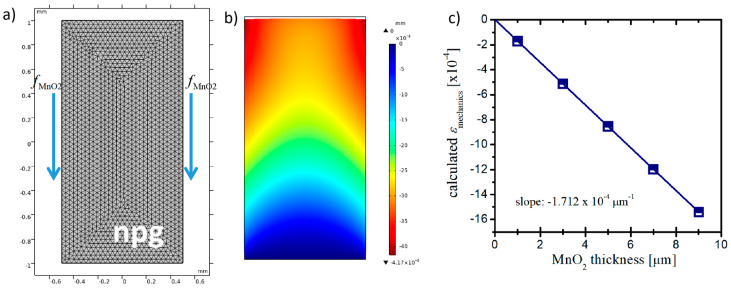
The finite element model of npg considering the mechanical effect (**a**); the simulated result for the length change of npg under the mechanical effect of MnO_2_ layer (**b**); the effect of MnO_2_ layer thickness on the actuation behavior of npg (**c**). The elastic modulus of the npg sample used in the simulation was 0.51 GPa from [45].

Liu et al. [37] investigated a reversible deformation of a freestanding MnO_2_/Ni bilayer film by in situ electrochemical atomic force microscopy. They found that the valence state variation of Mn element, shortening and lengthening of the Mn–O bond, and insertion and extraction of Na^+^ ions, led to the reversible contraction and expansion of MnO_2_ morphology. The change in distance between MnO_2_ micrometer particles at the surface was evaluated as ~2% within a 1 V potential window from the Atomic Force Microscope (AFM) data by Liu et al. [37]. The potential-induced expansion or contraction of the MnO_2_ layer results from the change in surface stress. A finite element model was built up to further understand our experimental data, as shown in Figure 6a. The simple FEM model in this paper introduced an additional force from the MnO_2_ layer on the outer surface of nanoporous gold and investigated the actuation behavior of npg with the MnO_2_ layer thickness. The mechanical effect of the MnO_2_ layer, *f*_MnO2_, can be roughly evaluated as:

(3) $$f_{{\mathrm{MnO}}_{2}}=-E_{{\mathrm{MnO}}_{2}}\varepsilon_{{\mathrm{MnO}}_{2}}h_{{\mathrm{MnO}}_{2}}$$

where *E*_MnO2_ is the elastic modulus of MnO_2_ (the value of 25 GPa was used in simulation from [44]); *ε*_MnO2_ is the potential-induced strain (we used the value of 1% in simulation since the potential interval was 0.5 V in our study); and *h*_MnO2_ is the thickness of the covered MnO_2_ layer. It could be seen that the thicker the MnO_2_ layer, the larger the contraction on npg.

Figure 6b exhibits the displacement distribution of npg under the electrochemical-induced force from MnO_2_ layer. The simulation results showed that the actuation amplitude of npg increased as the MnO_2_ thickness increased, as shown in Figure 6c. The best linear fit gave the slope value of −1.71 × 10^−4^ μm^−1^, which was quite close to the value obtained from the experimental measurement. This supports our conclusion that the MnO_2_ covering can significantly modulate the electrochemical actuation behavior of npg through the mechanical effect. The method in our work can then be used as a simple tool to improve or modulate the electrochemical actuation of other materials with nanoporous structures of not only the actuation amplitude, but also the actuation phase.

## 4. Conclusions

In summary, the electrochemical actuation performance of nanoporous gold was investigated with a focus on the effect of MnO_2_ layer. The electrochemical actuation of nanoporous gold was further enhanced with the covered MnO_2_ layer. The composite of npg and MnO_2_ showed an actuation amplitude of 9 × 10^−4^ at a potential scan rate of 1 mV s^−1^ with a thin MnO_2_ covering (~9 μm). Such a performance was significantly higher than that of the monolithic npg sample. Our work also found that the MnO_2_ layer inverted the response behavior of the sample to the applied potential (e.g., from expansion to contraction). This indicates that the response phase could be inverted from 0 degrees to 180 degrees. A simple finite element model in our study showed that the change in the amplitude and the phase of electrochemical actuation could be attributed to the contraction/expansion of MnO_2_ layer which is induced by the insertion and extraction of Na+ ions during electrochemical process. Compared to conventional strategies to improve the electrochemical actuation performance of nanoporous gold, our method is simple and more effective. Our results demonstrate that introducing a supercapacitive element into the electrochemical actuation system can be a powerful tool to modulate the activity of nanoporous materials.
